# Correlates and outcomes of preterm birth, low birth weight, and small for gestational age in HIV-exposed uninfected infants

**DOI:** 10.1186/1471-2393-14-7

**Published:** 2014-01-08

**Authors:** Jennifer A Slyker, Janna Patterson, Gwen Ambler, Barbra A Richardson, Elizabeth Maleche-Obimbo, Rose Bosire, Dorothy Mbori-Ngacha, Carey Farquhar, Grace John-Stewart

**Affiliations:** 1 Department of Global Health, University of Washington, Harborview Medical Center, 325 9th Ave, Box 359931, Seattle, WA 98104, USA; 2 Department of Pediatrics, Division of Neonatology, University of Washington, Seattle, USA; 3 Department of Medicine, Division of Allergy and Infectious Diseases, University of Washington, Seattle, USA; 4 Department of Biostatistics, University of Washington, Seattle, USA; 5 Department of Epidemiology, University of Washington, Seattle, USA; 6 Department of Paediatrics and Child Health, University of Nairobi, Nairobi, Kenya

**Keywords:** Preterm birth, Low birth weight, Small for gestational age, Pediatric HIV

## Abstract

**Background:**

Preterm birth (PTB), low birth weight (LBW) and small for gestational age (SGA) contribute to neonatal mortality. Maternal HIV-1 infection has been associated with an increased risk of PTB, but mechanisms underlying this association are undefined. We describe correlates and outcomes of PTB, LBW, and SGA in HIV-exposed uninfected infants.

**Methods:**

This was a retrospective analysis of cohort study. Between 1999–2002, pregnant, HIV-infected women were enrolled into an HIV-1 transmission study. Logistic regression was used to identify correlates of PTB, LBW and SGA in HIV-negative, spontaneous singleton deliveries. Associations between birth outcomes and mortality were measured using survival analyses.

**Results:**

In multivariable models, maternal plasma (OR = 2.1, 95% CI = 1.1-3.8) and cervical HIV-1 RNA levels (OR = 1.6, 95% CI = 1.1-2.4), and CD4 < 15% (OR = 2.4, 95% CI = 1.0-5.6) were associated with increased odds of PTB. Abnormal vaginal discharge and cervical polymorphonuclear leukocytes were also associated with PTB. Cervical HIV-1 RNA level (OR = 2.4, 95% CI = 1.5-6.7) was associated with an increased odds of LBW, while increasing parity (OR = 0.46, 95% CI = 0.24-0.88) was associated with reduced odds. Higher maternal body mass index (OR = 0.75, 95% CI = 0.61-0.92) was associated with a reduced odds of SGA, while bacterial vaginosis was associated with >3-fold increased odds (OR = 3.2, 95% CI = 1.4-7.4). PTB, LBW, and SGA were each associated with a >6-fold increased risk of neonatal death, and a >2-fold increased rate of infant mortality within the first year.

**Conclusions:**

Maternal plasma and cervical HIV-1 RNA load, and genital infections may be important risk factors for PTB in HIV-exposed uninfected infants. PTB, LBW, and SGA are associated with increased neonatal and infant mortality in HIV-exposed uninfected infants.

## Background

Globally, it is estimated that there are more than 13 million preterm births (PTB) annually, comprising 11% of live births, with the highest burden in low-income countries [1,2]. PTB is both a direct and indirect leading cause of mortality in the neonatal period, accounting for 28% of global neonatal deaths [3]. Low birth weight (LBW) and small for gestational age (SGA) newborns are also at elevated risk for death (reviewed in [4]). Beyond the neonatal period, preterm infants are at an increased risk of morbidity and mortality, developmental delays, lower educational attainment, and increased lifetime risk of non-communicable diseases [5].

Mechanisms resulting in PTB are not fully understood. Maternal infection, and vaginal infection in particular, is thought to be a major pathway to PTB [6,7]. Markers of inflammation and infection have been associated with PTB, and treatment for some vaginal infections has been shown to reduce the rates of PTB [7,8]. To date, a handful of studies have examined correlates of adverse birth outcomes in the setting of maternal HIV-1 infection, with variable findings. Maternal plasma HIV-1 RNA load, CD4 counts and symptomatic disease have been associated with PTB in some studies but not others [9-11]. Differences in sociodemographics, behavior, genetics, vaginal flora, co-infections, secular time, and delivery inclusion/exclusion criteria (gestational age cutoff, elective caesarean sections and twins), undoubtedly result in confounding, which could attenuate observed associations with PTB.

Maternal antiretroviral therapy (ART) for either prevention of mother to child transmission (PMTCT) or treatment has introduced further complexity to discerning relationships between HIV-1 and birth outcomes. The risk of PTB declined in HIV-infected women during the period between 1989–2004 in the United States [12], in contrast to increasing rates of PTB in the general population during the same period [13], suggesting that virologic suppression and/or immune reconstitution likely improves birth outcomes on the whole. However, some studies have shown differences in PTB rates by maternal ART regimen, protease-inhibitor use, or timing of ART initiation, suggesting specific drugs, or drug classes may increase the risk of PTB [14,15]. Current evidence suggests the use of protease-inhibitor (PI)-based regimens may increase the risk of PTB over that of non-PI-based regimens [16,17].

As ART use expands in sub-Saharan Africa, the population of HIV-1 exposed uninfected infants continues to grow. HIV-exposed uninfected infants have increased rates of morbidity and mortality compared to unexposed infants (reviewed in [18]). The reason for increased morbidity among HIV-exposed uninfected infants is likely due to a combination of factors including reduced maternal transfer of antibodies, increased exposure to infectious pathogens from their mother, and altered immunologic development. Studies to better understand the vulnerability of HIV-exposed uninfected children will enable the development of strategic interventions to optimize outcomes in this unique population. Utilizing data from an historic perinatal cohort, we estimated the incidence of PTB, LBW, and SGA in a cohort of HIV-infected women and their uninfected infants. We additionally determined correlates of PTB, LBW and SGA; and quantified their association with infant mortality during the first year of life.

## Methods

### Study population

Protocols were approved by the Institutional Review Board of the University of Washington and the Ethics and Research Committee of Kenyatta National Hospital. HIV-infected women with gestation ≥28 weeks were recruited from Nairobi antenatal clinics from 1999-2002 [19]. Fetal gestation at enrollment was estimated by a combination of last menstrual period and fundal height. Women received short-course zidovudine for PMTCT [19].

### Clinical assessments and follow-up

Medical and obstetric histories were recorded at enrollment. At 32 weeks, clinicians performed a pelvic examination and collected blood and genital specimens for sexually transmitted infection (STI) screening [20]. *Trichomonas vaginalis* was diagnosed from vaginal swabs by in-pouch culture testing using the APTIMA platform (Gen-Probe, San Diego, California). Cervical swabs were used for *Neisseria gonorrhoeae* and *Chlamydia trachomatis* testing using the Amplicor CT/NG test (Roche Molecular Systems Inc, Branchburg, New Jersey). *Treponema pallidum* was diagnosed by the rapid plasma reagin test (Becton and Dickinson, Franklin Lakes, New Jersey) with confirmation by Treponema pallidum haemagglutination assay (Randox Laboratories Ltd, Crumlin, UK). Bacterial vaginosis (BV) was diagnosed using Nugent criteria from Gram-stained vaginal smears and *Candida* was identified by visualization of vaginal wet mount. Sexually transmitted and vaginal infections were treated as indicated. Mothers returned monthly for interviews and clinical assessments.

Neonates were examined at birth by a study physician. For deliveries outside the study site, birth weight data was abstracted from facility records or government-issued mother-child health booklets. Infant gestational age at birth was assessed via Dubowitz scoring [21]. In the absence of Dubowitz, last menstrual period was used to estimate gestational age for overall incidence estimates. Mother-infant pairs attended monthly visits in the clinic for one year. For deceased infants, age at death was determined by a physician following a chart review and/or verbal autopsy with the parent or guardian [19,22].

### Definitions

Infants were considered preterm births (PTB) if born before 37 weeks. Low birth-weight (LBW) was defined as less than 2.5 kg. To account for early neonatal weight loss, weight data were only included from infants weighed within 24 hours of birth. Similarly, analysis of Dubowitz assessments was restricted to those conducted within 3 days of birth. Small for gestational age (SGA) was determined via the method outlined by Mikolajczyk [23] utilizing the mean birth weight at 40 weeks in our cohort and the standard deviation specific to our sample, and using Dubowitz-estimated gestational age. Neonatal and infant mortality were defined as deaths occurring within the first 28 days, and 365 days of life, respectively.

### HIV-1 testing and viral loads

HIV-1 RNA loads were measured using the GenProbe assay [24] in plasma and cervical swabs at 32 weeks, and in maternal plasma at delivery. HIV-1 testing was performed on infant blood at birth (<48 hours) and 1, 3, 6, 9 and 12 months. Infant HIV-1 infection was defined by the detection of HIV-1 DNA in dried blood spots [25] or RNA in plasma [24]. Uninfected infants received a confirmatory HIV-1 ELISA at study exit.

### Statistical methods

Stata SE v11.2 for Macintosh (StataCorp, College Station, Texas) was used for all analyses. All tests were two-tailed with alpha = 0.05. Overall estimates of PTB rates included deliveries where either Dubowitz or last menstrual period was available. Fisher’s exact test was used to compare the proportion of PTBs between infants with and without HIV-1 detection at birth.

Analyses for correlates of adverse birth outcomes and mortality were limited to spontaneous deliveries of singleton, HIV-uninfected infants. We excluded twins (n = 7 sets), planned cesarean sections (n = 20), infants who were HIV-infected at birth (n = 29), and those who lacked an HIV-1 test at birth (1 intrapartum death and 7 stillbirths). When analyzing correlates of PTB, we used only infants with Dubowitz assessment, because this method is more reliable than last menstrual period when compared to ultrasound [26].

Logistic regression was used to identify correlates of PTB, LBW and SGA. Covariates included *a priori* defined variables based on literature review and hypothesized relationships between maternal HIV-1 and birth outcome. In order to generate meaningful estimates, we required a minimum of 10 exposures for each covariate included in regression models; for this reason several well-defined risk factors (smoking, pre-eclampsia, eclampsia, *Neisseria gonorrhoeae*) were not evaluated because they were uncommon in this cohort (numbers are provided in Table 1). We used stepwise logistic regression to create final multivariable models; for each of the three outcomes we included all covariates with p values ≤0.1, then performed backward elimination of covariates until we had a best-fit model. Pearson’s correlation was used to measure the correlation between plasma and cervical HIV-1 RNA loads; these terms were collinear so they were not included together in any multivariable models and we present alternative models including each separately. Similarly, we made separate models for either viral load or CD4 because these terms were also collinear. In multivariable analyses, we elected to adjust for CD4 percent as an indicator of immunosuppression, rather than CD4 count, because CD4 percent is not affected by increasing blood volume during pregnancy, which causes a decline in CD4 cells/mm^3^.

**Table 1:** Population characteristics of HIV-infected women spontaneously delivering singleton, HIV-uninfected infants

	**N**	**Median (IQR) or proportion (n)**
Sociodemographic		
Married	413	90% (372)
Employed	413	30% (123)
Years education	408	8 (8–11)
Physical and Obstetric (Current pregnancy)		
Age (years)	413	25 (22–28)
Body mass index at 32 weeks	400	24 (23–26)
Parity	409	1 (1–2)
Pre-eclampsia	408	1.5% (6)
Eclampsia	407	0.25% (1)
Place of delivery		
Kenyatta National Hospital	412	85% (350)
Other facility	412	6.1% (25)
Home	412	7.5% (31)
En route to hospital	412	1.5% (6)
Caesarean section	394	15% (58)
Alcohol use	410	3.9% (16)
Cigarette smoking	412	1.2% (5)
Vaginal infections at 32 weeks		
Bacterial vaginosis	385	37% (144)
*Candida*	408	30% (124)
Cervical PMN ≥3 per high-powered field	336	61% (204)
Abnormal vaginal discharge	408	50% (204)
Cervical blood	408	4.7% (19)
*Trichomonas vaginalis*	408	16% (65)
*Chlamydia trachomatis*	407	3.9% (16)
*Treponema pallidum*	409	2.4% (10)
*Neisseria gonorrhoeae*	407	2.0% (8)
Any sexually transmitted infection^a^	413	21% (88)
Immunologic and virologic		
CD4 cells/mm^3^ <350	401	34% (137)
CD4 percent <15	401	15% (60)
Log_10_ plasma HIV-1 RNA load	396	4.7 (4.2-5.2)
Log_10_ cervical HIV-1 RNA load	315	2.0 (1.4-2.9)
Cervical HIV DNA detectable	315	72% (227)
Delivery		
Baby female	412	46% (191)
Dubowitz estimated maturity (weeks)	335	40 (39–41)
Premature	335	9.9% (33)
Dubowitz estimated maturity in preterm (weeks)	33	35 (34–36)
Low birth weight	332	6.0% (20)
Birth weight in low birth weight infants (kg)	332	2.2 (1.5-2.4)
Small for gestational age	311	9.0% (28)

Notes. All clinical assessments and laboratory measurements were performed at 32 weeks gestation. ^a^Includes laboratory-confirmed *Neisseria gonorrhoeae, Trichomonas vaginalis, Chlamydia trachomatis,* and *Treponema pallidum*.

Mortality rates are reported separately for the neonatal (28 day) and infant (365 day) periods. All spontaneously-delivered infants born HIV-negative were considered at risk from birth; we excluded from this analysis infants who acquired HIV-1 later during follow-up. Kaplan-Meier survival analysis was used to estimate the neonatal mortality incidence rate; surviving children were censored at 28 days. For infant mortality, individuals were censored at study exit, or 1 year, whichever came first. Cox proportional hazards regression was used to estimate hazard ratios (HR) for PTB, LBW, and SGA, individually, and adjusted for maternal plasma HIV-1 RNA load at 32 weeks gestation, with censoring at study exit or 1 year, whichever came first. Kaplan-Meier survival plots with the log-rank statistic are provided to illustrate differences between mortality in the different groups over time.

## Results

### Overall rate of preterm birth

A total of 475 women delivered 482 infants. Gestational age was estimated using Dubowitz (385 women) or last menstrual period (80 women) in 465 women. The overall PTB rate was 14% (65/465). PTB comprised 28% (8/29) of deliveries of HIV-infected infants (inclusive of 2 twin deliveries), and 13% (57/429) of HIV-uninfected infants (inclusive of 5 twin deliveries, p = 0.05).

### Participant characteristics

**Figure 1:**
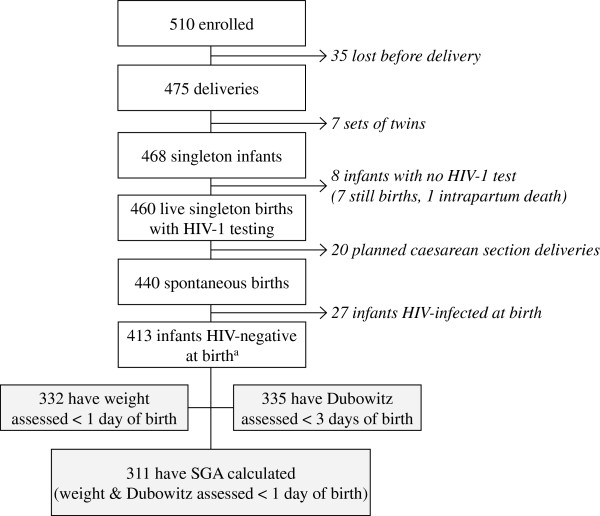
Patient flow chart.

A total of 413 women spontaneously delivered live, singleton infants who were HIV-negative at birth (Figure 1); 332 infants (80%) had weight assessed within the first day, 335 (81%) had Dubowitz assessed within 3 days, and 311 (75%) had both Dubowitz and weight assessed within the specified time limits enabling calculation of SGA. Of the 413 infants born HIV-negative, 54 (13%) later acquired HIV-1.

Characteristics of the women who spontaneously delivered live, singleton HIV-negative infants are detailed in Table 1. Most mothers were young and married. The majority of deliveries occurred at the study site, Kenyatta National Hospital (85%). Cigarette smoking alcohol use, pre-eclampsia and eclampsia were rare. Vaginal infections were common; 37% of women had BV, 30% had *Candida*, and 21% had a laboratory-confirmed STI at 32 weeks gestation.

### Rates of PTB, LBW and SGA in spontaneously delivered, singleton, HIV-uninfected infants

There were 33 spontaneous, singleton preterm deliveries of HIV-exposed uninfected infants (9.9%). The median gestational age of PTB infants was 35 weeks, (IQR = 34-36). Of the 29 preterm infants with birth weight assessed within 24 hours, 11 (38%) were also LBW and 3 were SGA (10%). A total of 6.0% (20/332) of infants were LBW. A large proportion of LBW cases were explained by preterm delivery, 61% (11/18). Overall, 9.0% of infants were SGA (28/311); 11% of these SGA infants had been born preterm.

### Correlates of PTB, LBW and SGA among spontaneous, singleton HIV-negative deliveries

Maternal cervical HIV-1 RNA load (odds ratio (OR) = 1.8, p = 0.001), plasma HIV-1 RNA load (OR = 1.9, p = 0.02), and CD4 percent < 15 (OR = 3.3, p = 0.004) were associated with an increased odds of PTB (Table 2). No specific STIs or genital infections were significantly associated with PTB. However, abnormal vaginal discharge (OR = 2.4, p = 0.03), detection of cervical polymorphonuclear leukocytes (PMN) at a density of ≥3 per high-power field (the cohort median; OR = 3.2, p = 0.02), and detection of blood in the cervix (OR = 5.1, p = 0.002) were associated with an increased odds of PTB. There was a non-significant increased odds of PTB in women with BV diagnosed at 32 weeks (OR = 2.1, p = 0.06).

**Table 2:** Correlates of prematurity, low birth weight and small for gestational age deliveries of HIV-exposed uninfected infants

	**Preterm**		**Low birth weight**		**Small for gestational age**	
A) Univariable analysis	OR [95% CI]	P	OR [95% CI]	P	OR [95% CI]	P
Female infant	1.1 [0.53-2.3]	0.8	2.3 [0.88-5.8]	0.09	2.7 [1.2-6.2]	0.02
Married	1.8 [0.42-8.0]	0.4	0.65 [0.18-2.3]	0.5	0.88 [0.25-3.1]	0.8
Employed	0.87 [0.39-1.9]	0.7	1.0 [0.39-2.8]	0.9	1.0 [0.42-2.4]	>0.9
Years education	0.89 [0.78-1.0]	0.1	1.1 [0.94-1.3]	0.2	1.1 [0.96-1.3]	0.1
Age (years)	0.97 [0.89-1.1]	0.5	0.93 [0.83-1.0]	0.2	0.97 [0.89-1.1]	0.5
Body mass index	0.90 [0.78-1.0]	0.1	0.74 [0.61-0.91]	0.004	0.80 [0.68-0.94]	0.007
Parity	0.91 [0.68-1.2]	0.6	0.52 [0.30-0.89]	0.02	0.78 [0.53-1.1]	0.2
Alcohol use	^b^		^c^		1.7 [0.37-8.2]	0.5
Bacterial vaginosis	2.1 [0.97-4.4]	0.06	2.1 [0.78-5.6]	0.1	3.2 [1.4-7.3]	0.005
*Candida*	0.90 [0.40-2.0]	0.8	1.1 [0.41-3.1]	0.8	0.74 [0.30-1.8]	0.5
Cervical PMN ≥3 per high-powered field^a^	3.2 [1.2-8.9]	0.02	2.9 [0.81-10]	0.1	1.9 [0.75-4.7]	0.2
Abnormal vaginal discharge	2.4 [1.1-5.2]	0.03	1.8 [0.70-4.7]	0.2	0.89 [0.41-1.9]	0.8
Cervical blood	5.1 [1.8-14]	0.002	2.5 [0.52-12]	0.3	0.65 [0.083-5.1]	0.7
*Trichomonas vaginalis*	0.64 [0.19-2.2]	0.5	0.32 [0.042-2.5]	0.3	0.72 [0.21-2.5]	0.6
*Chlamydia trachomatis*	1.7 [0.35-7.8]	0.5	3.4 [0.69-17]	0.1	0.82 [0.10-6.6]	0.9
*Treponema pallidum*	^b^		1.8 [0.21-15]	0.6	1.1 [0.14-9.2]	0.9
Any sexually transmitted infection	0.68 [0.25-1.8]	0.4	0.91 [0.30-2.8]	0.9	0.81 [0.29-2.2]	0.7
CD4 cells/mm^3^ <350	1.9 [0.90-4.0]	0.09	2.0 [0.79-5.3]	0.1	1.1 [0.50-2.5]	0.8
CD4 percent <15	3.3 [1.4-7.7]	0.004	1.8 [0.58-5.9]	0.3	1.0 [0.33-3.1]	>0.9
Log_10_ HIV plasma HIV-1 RNA load at 32 weeks	1.9 [1.1-3.1]	0.02	1.5 [0.81-2.9]	0.2	1.1 [0.69-1.8]	0.7
Log_10_ HIV cervical HIV-1 RNA load at 32 weeks	1.8 [1.3-2.5]	0.001	2.1 [1.4-3.2]	<0.001	1.3 [0.88-1.8]	0.2
	**Preterm**		**Low birth weight**		**Small for gestational age**	
B) Multivariable analyses	OR [95% CI]	P	OR [95% CI]	P	OR [95% CI]	P
*Model 1 (includes plasma HIV-1 RNA load)*						
Cervical PMN ≥3 per high-powered field	2.9 [1.2-7.3]	0.02	*		*	
Abnormal vaginal discharge	3.1 [1.2-7.9]	0.02	*		*	
Log_10_ HIV plasma HIV-1 RNA load	2.1 [1.1-3.8]	0.03	*		*	
*Model 2 (includes cervical HIV-1 RNA load)*						
Cervical PMN ≥3 per high-powered field	2.7 [1.0-7.3]	0.05	*		*	
Abnormal vaginal discharge	3.6 [1.2-10]	0.02	*		*	
Log_10_ HIV cervical HIV-1 RNA load	1.6 [1.1-2.4]	0.02	*		*	
*Model 3 (includes CD4 percent <15)*^*d*^						
Cervical PMN ≥3 per high-powered field	3.5 [1.4-8.6]	0.007	*		*	
Abnormal vaginal discharge	3.2 [1.3-8.2]	0.01	*		*	
CD4 percent <15	2.4 [1.0-5.6]	0.05	*		*	
*Model 4*						
Parity	*		0.46 [0.24-0.88]	0.02	*	
Log_10_ HIV cervical HIV-1 RNA load	*		2.4 [1.5-6.7]	<0.001	*	
*Model 5*						
Body mass index	*		*		0.75 [0.61-0.92]	0.005
Bacterial vaginosis	*		*		3.2 [1.4-7.4]	0.007

Notes. All clinical assessments and laboratory measurements were performed at 32 weeks gestation. ^a^Cohort median for polymorphonuclear cells (PMN) detected was 3. ^b^No women who used alcohol during pregnancy, and no women with *Treponema pallidum* delivered preterm. ^c^No women who used alcohol during pregnancy delivered a low birth weight infant. B) Multivariable logistic regression models. Stepwise logistic regression was used to create final multivariable models for each of the three outcomes as described in the Statistical Methods; because plasma HIV viral load, cervical HIV viral load, and CD4 percent were collinear, we adjusted for these in separate models. ^*d*^An alternative model including CD4 < 350 cells/mm^3^ returned point estimates for PMN and vaginal discharge that were similar to Model 3 (data not shown). * indicates that this outcome was not assessed in the model.

Correlates of LBW included cervical HIV-1 replication at 32 weeks gestation; each 1-log increase in cervical HIV-1 RNA load was associated with a >2-fold increased odds of LBW (OR = 2.1, p < 0.001). Higher maternal body mass index (BMI, OR = 0.74, p = 0.004) at 32 weeks gestation and increasing parity (OR = 0.52, p = 0.02) were associated with reduced odds of LBW.

Unlike PTB and LBW, no maternal immune or virologic indicators were significantly associated with SGA. SGA was more common in female infants (OR = 2.7, p = 0.02), and in the presence of maternal BV (OR = 3.2, p = 0.005). Higher BMI at 32 weeks gestation was associated with reduced odds of SGA (OR = 0.80, p = 0.007).

### Adjusted correlates of PTB, LBW and SGA among spontaneous, singleton HIV-negative deliveries

The odds of PTB were independently associated with cervical PMN detection (OR = 2.9, 95% CI = 1.2-7.3; p = 0.02; Model 1), abnormal vaginal discharge (OR = 3.1, 95% CI = 1.2-7.9; p = 0.02; Model 1), log_10_ plasma HIV-1 RNA load (OR = 2.1, 95% CI = 1.1-3.8; p = 0.03; Model 1), log_10_ cervical HIV-1 RNA load (OR = 1.6, 95% CI = 1.1-2.4; p = 0.02; Model 2), and CD4% less than 15% (OR = 2.4, 95% CI = 1.0-5.6; p = 0.05; Model 3). The odds of LBW (Model 4) were independently associated with log_10_ plasma HIV-1 RNA load (OR = 2.4, 95% CI = 1.5-6.7; p < 0.001), whereas each additional live birth in a woman’s history was associated with reduced odds of LBW (OR for parity = 0.46, 95% CI = 0.24-0.88; p = 0.02). BV was associated with a >3-fold increased odds of SGA (Model 5; OR = 3.2, 95% CI = 1.4-7.4; p = 0.007), whereas each 1-unit increase in maternal BMI at 32 weeks gestation was associated with a 25% reduced odds of SGA (OR = 0.75, 95% CI = 0.61-0.92; p = 0.005).

### PTB, LBW, SGA and mortality

From the subset of 413 infants born HIV-negative, 359 remained HIV-uninfected until study exit or death; of these 287 had Dubowitz assessed within 72 hours of birth, 287 had birth weight assessed within 24 hours of birth, and 268 had SGA evaluated (both Dubowitz and birth weight assessed in 48 hours).

A total of 32 infant deaths were recorded in the HIV-exposed uninfected infants during the first year of life, and 13 occurred during the first 28 days of life. The neonatal mortality incidence rate (IR) for infants born PTB was 7-fold higher than infants born at term (IR = 5.8 vs 0.81 per 1000 person-days, incidence rate ratio (IRR) = 7.1, 95% CI = 1.5-30, p = 0.008). The neonatal mortality IR for infants born LBW was more than 6-fold higher than infants born at normal weight (IR = 6.7 versus 1.0 per 1000 person-days, IRR = 6.4, 95% CI = 1.1-27, p = 0.02). The neonatal mortality IR for infants born SGA was 7-fold higher than infants born with normal weight for gestational age (5.4 versus 0.73 per 1000 person-days, IRR = 7.4, 95% CI = 1.5-34, p = 0.009).

**Figure 2:**
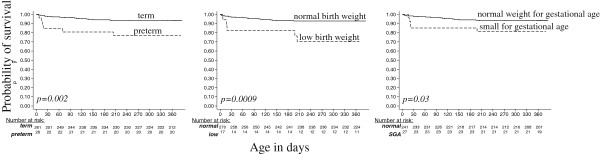
**Survival of preterm, low birth weight, and small for gestational age HIV-exposed uninfected infants.** Curves show survival functions for infants grouped by prematurity, low birth weight, and small for gestational age. P values are from log-rank test.

We also examined associations between PTB, LBW and SGA and infant survival during the first year of life. In unadjusted analyses, PTB, LBW, SGA, and maternal prenatal HIV-1 RNA load were all associated with an increased rate of infant death (Table 3 & Figure 2). When adjusting for maternal plasma HIV-1 RNA load at 32 weeks gestation, PTB (hazard ratio (HR) = 2.7, p = 0.05), LBW (HR = 3.3, p = 0.03) and SGA (HR = 2.9, p = 0.04) remained independently associated with time to infant death.

**Table 3:** Correlates of infant mortality during 12-month follow-up

	**Unadjusted**			**Adjusted for maternal plasma HIV-1 RNA load**		
	**N**	**HR [95% CI]**	**P value**	**N**	**aHR [95% CI]**	**P value**
Preterm birth	287	3.9 [1.5-10]	0.004	275	2.7 [1.0-7.5]^b^	0.05
Low birth weight	287	4.5 [1.7-12]	0.003	277	3.3 [1.1-10]^c^	0.03
Small for gestational age	268	3.0 [1.1-8.1]	0.03	258	2.9 [1.0-8.0]^d^	0.04
Maternal log_10_ HIV plasma HIV-1 RNA load ^a^	342	1.8 [1.1-2.9]	0.03		^b, c, d^	

Notes. Includes only children remaining HIV-uninfected throughout follow-up. Cox regression, censored at 1 year. Each outcome was adjusted separately for maternal log_10_ plasma HIV-1 RNA load. ^a^Viral load measured at 32 weeks gestation. ^b^aHR = 1.6, p = 0.2 for HIV-1 RNA load. ^c^aHR = 1.2, p = 0.5 for HIV-1 RNA load. ^d^aHR = 1.4, p = 0.3 for HIV-1 RNA load.

## Discussion

This study identified several potentially modifiable factors that were associated with PTB, LBW, and SGA among HIV-exposed uninfected infants. Maternal genital infection was a predominant correlate of PTB in this cohort: cervical HIV-1 RNA load, the detection of PMN in cervical fluid, and vaginal discharge were all associated with increased odds of PTB. Cervical HIV-1 RNA load was also independently associated with an increased likelihood of delivering a LBW infant. BV and maternal BMI in pregnancy were independently associated with SGA, but HIV-1 RNA load was not. The consequences of being born preterm or small were profound; PTB, LBW and SGA were each significantly associated with >6-fold increased neonatal mortality rate and a >2-fold increased infant mortality rate. These data demonstrate that PTB, LBW, and SGA may be associated with mortality in HIV-exposed uninfected children, and suggest that reduction of maternal genital HIV-1 replication could be a strategy to reduce the risk of late preterm birth and low birth weight deliveries.

Genital infection/inflammation, as indicated by vaginal discharge and PMN in cervical swabs, emerged as important predictors of PTB and LBW in this cohort. To our knowledge, this is the first report demonstrating an association between HIV-1 RNA load in cervical fluid and PTB or LBW. Ascending infection into the uterus is one mechanism by which lower genital infections are thought to trigger PTB [27]. Additionally, the process of cervical ripening could be accelerated by HIV-induced inflammation and migration of leukocytes to infected cervical tissue. Cervicitis has been associated with PTB and/or LBW delivery in several studies [28,29], and a study by Brown and colleagues reported an association between the detection of cervical HSV-2 shedding and PTB [30]. Together, these data are consistent with the evolving model of PTB that includes maternal genital infection as a primary pathway leading to PTB (discussed in [27,31]). Whether any of these associations are causal in nature is beyond the scope of our study, however, they point toward reduction of cervical infection or inflammation as potential interventions to reduce the risk of PTB.

The overall rate of PTB (14%) in this cohort of HIV-infected women was similar to general population estimates for Kenya in 2010 (10-15% [1]). Although HIV-infected women have been shown to be at an increased risk of PTB in research studies, population rates of PTB in HIV-infected women may be counterbalanced by their overall reduced fertility and increased mortality. Additionally, because women were enrolled after 28 weeks, extremely preterm deliveries were not measured, which could explain the low rate of PTB observed in this cohort. Consistent with previous studies, we found a higher rate of PTB in infants born HIV-infected compared to those born HIV-uninfected [12,32]. Maternal HIV-1 RNA load, CD4 count, and/or advanced HIV-1 disease have been associated with PTB in previous studies [10,11]. Similarly, we observed independent associations between maternal plasma HIV-1 RNA load, immunosuppression and PTB. Because plasma HIV-1 RNA load, cervical HIV-1 RNA load, and immunosuppression are strongly correlated with one another, is difficult to determine the potential contribution of each to PTB.

LBW deliveries were associated with maternal weight, BMI, and parity, consistent with previous reports in both HIV-infected and uninfected populations [33,34]. In a study of HIV-infected women from Mombasa, Kenya correlates of LBW included age, education, primiparity, prior neonatal death, and delivery of a female infant [35]. Maternal HIV-1 RNA load and/or CD4 count have also been associated with LBW, intrauterine growth restriction, or SGA [9,11,34]. In our multivariable analyses, only parity and cervical HIV-1 RNA load were independently associated with LBW. The association between LBW and cervical HIV-1 RNA load could be explained by the large degree of overlap between PTB and LBW infants; this is somewhat supported by the observation that SGA was not associated with any maternal virologic indicators. SGA was associated with delivery of a female infant, BMI, and BV, which is consistent with previous studies in HIV-negative women [36,37].

Globally, PTBs account for the majority of deaths in the neonatal period [3]. A recent meta-analysis of studies in East Africa reported high odds ratios for death before 28 days for PTB (OR = 6.2), moderate LBW (OR = 6.2), and SGA (2.1) compared to normal infants [38]. Similarly, neonatal mortality rates were at least 6-fold higher for PTB, LBW, and SGA infants in our study. Among HIV-exposed uninfected infants who escaped postnatal infection, PTB, LBW, and SGA delivery were also associated with an increased risk of infant mortality during the first year of life. Causes of death in the HIV-exposed uninfected infants from this cohort have been detailed elsewhere [22]; most commonly involved were sepsis, pneumonia and failure to thrive.

Our study has many strengths and some limitations. Weight, gestation, and mortality were systematically assessed. Most deliveries occurred at a facility, and Dubowitz assessments of gestational age were conducted by physicians. Since the goal of our study was to identify modifiable maternal factors that could be targeted to reduce the risk of PTB and LBW deliveries, we limited analyses to spontaneous, singleton deliveries of HIV-uninfected infants, to reduce confounding and increase sensitivity to detect virologic associations. Additionally, we only included infants with Dubowitz assessment to reduce misclassification of outcome. Although these data are a decade old, this cohort was accrued during a time when antiretroviral treatment was only rarely available in Kenya; we were therefore able to look at correlates of birth outcomes in a setting unconfounded by combination maternal antiretroviral therapy. Sociodemographic homogeneity of the cohort, and the rarity of well-defined risk factors such as smoking, drinking and illicit-drug use also reduced confounding and increased our sensitivity to detect virologic associations with birth outcomes. However, ultrasound dating was not available, limiting precision of gestational age estimates. Finally, current Option B and B-plus [39] PMTCT regimens involve maternal combination ART which may result in different PTB estimates and correlates than we observed in our setting of short-course zidovudine. As Option B-plus expands treatment to a larger population of women, it will be important to understand the effect of maternal HAART, and different HAART regimens, on the risk of adverse birth outcomes.

## Conclusions

Our data support the association between maternal genital infection and preterm delivery in HIV-exposed uninfected infants. As the risks and benefits of increasingly efficacious PMTCT regimens and earlier PMTCT initiation are weighed, our data suggest that reduction of cervical viral replication could potentially reduce the risk of PTB and LBW deliveries. Additionally, HIV-exposed uninfected infants born PTB, LBW, or SGA are at an elevated risk for death during the neonatal and infant periods, and warrant heightened clinical monitoring.

## Competing interests

The authors declare that they have no competing interests.

## Authors’ contributions

All authors have contributed to the preparation of this manuscript and have approved this final version. JAS and JP conducted the analyses and co-wrote the paper. BAR and GA contributed to study design, the statistical analysis plan, and determination of the final statistical models used. EM-O, RB, CF and DM-N developed the clinical protocols, birth outcome definitions, and executed the cohort study. GJS was PI of the project study that accrued the cohort, and participated in development of the manuscript aims, analyses, and interpretation of results.

## Pre-publication history

The pre-publication history for this paper can be accessed here:

http://www.biomedcentral.com/1471-2393/14/7/prepub

## Conference presentation

Presented as a poster at the 6^th^ IAS Conference on HIV Pathogenesis, Treatment and Prevention, Rome, Italy 2011.
